# Mammalian Argonaute-DNA binding?

**DOI:** 10.1186/s13062-014-0027-4

**Published:** 2014-12-04

**Authors:** Neil R Smalheiser, Octavio L A Gomes

**Affiliations:** Department of Psychiatry and Psychiatric Institute, University of Illinois at Chicago, 1601 W. Taylor Street MC912, Chicago, IL 60612 USA

**Keywords:** Consensus, Hypothesis assessment, Scientific discovery, DNA interference, Cytoplasmic DNA, RNA interference

## Abstract

When a field shares the consensus that a particular phenomenon does NOT occur, this may reflect extensive experimental investigations with negative outcomes, or may represent the “common sense” position based on current knowledge and established ways of thinking. The current consensus of the RNA field is that eukaryotic Argonaute (Ago) proteins employ RNA guides and target other RNAs. The alternative -- that eukaryotic Ago has biologically important interactions with DNA in vivo – has not been seriously considered, in part because the only role contemplated for DNA was as a guide strand, and in part because it did not seem plausible that any natural source of suitable DNAs exists in eukaryotic cells. However, eukaryotic Argonaute domains bind DNA in the test tube, and several articles report that small inhibitory double-stranded DNAs do have the ability to silence target RNAs in a sequence-dependent (though poorly characterized) manner. A search of the literature identified potential DNA binding partners for Ago, including (among others) single-stranded DNAs residing in extracellular vesicles, and cytoplasmic satellite-repeat DNA fragments that are associated with the plasma membrane and transcribed by Pol II. It is interesting to note that both cytoplasmic and extracellular vesicle DNA are expressed at greatly elevated levels in cancer cells relative to normal cells. In such a pathological scenario, if not under normal conditions, there may be appreciable binding of Ago to DNA despite its lower affinity compared to RNA. If so, DNA might displace Ago from binding to its normal partners (miRNAs, siRNAs and other short ncRNAs), disrupting tightly controlled post-transcriptional gene silencing processes that are vital to correct functioning of a normal cell. The possible contribution to cancer pathogenesis is a strong motivator for further investigation of Ago-DNA binding. More generally, this case underscores the need for better informatics tools to allow investigators to analyze the state of a given scientific question at a high-level and to identify possible new research directions.

**Reviewers:** This article was reviewed by Eugene Koonin, Kira S. Makarova, Alexander Maxwell Burroughs *(nominated by L Aravind)*, and Isidore Rigoutsos.

**Open peer review:** Reviewed by Eugene Koonin, Kira S. Makarova, Alexander Maxwell Burroughs *(nominated by L Aravind)*, and Isidore Rigoutsos. For the full reviews, please go to the Reviewers’ comments section.

## Background

Analyses of new emerging scientific research trends are almost always done retrospectively, rather than prospectively. Yet we can only assert that we understand a phenomenon if we can predict its occurrence at a given time and place in the future. How might we recognize situations in which a new trend has not yet started? It is one thing to predict the Beatles will be stars after they have appeared on the Ed Sullivan show, quite another to do so while they are playing in Hamburg.

One possible strategy is to look for fields in which new enabling methods and tools have just been introduced. Another is to focus on situations in which there is great activity and great turmoil – e.g., TAUists vs. BAPtists in Alzheimer disease, or proteins vs. nucleic acids in prion disease -- where the scientific community openly discusses, and attempts to reconcile, controversies based on disparate observations or experimental analyses. However, we suggest that new trends may also be situated in the absence of controversy: Namely, when the mainstream of a field shares the consensus that a particular phenomenon does NOT occur, and papers stating the negative consensus do not feel that it is necessary to provide any detailed arguments, documentation or citations to back up the assertion. This is a sign that the negative consensus is not the result of intensive experimental investigations that tested and ruled out the phenomenon exhaustively, but rather represents the “common sense” position based on current knowledge and established ways of thinking. Since these might readily be overturned in the light of new empirical findings, they represent situations in which a small input of new data might have a large output in terms of new lines of investigation. This is somewhat like predicting forest fires by identifying areas that are experiencing very dry conditions – nothing at all has happened yet, but a single match could spread flames like – well, like wildfire!

The starting point for our analysis is the publication of two breakthrough papers (Olovnikov et al. [1]; Swarts et al. [2]), which provided experimental validation of predictions made previously by Makarova et al. [3], namely, that prokaryotic Argonaute proteins are able to bind DNA and to carry out DNA-guided as well as RNA-guided mediated cleavage of DNA molecules (“DNA interference”) [1–3]. This appears to provide a form of protection of a bacterial cell against foreign invading plasmids and possibly other sources of foreign DNA. We were intrigued that both papers, as well as numerous following news-and-views articles, all stated or implied that eukaryotic Argonaute proteins only interact with RNA molecules and not with DNA. For example: “eukaryotic Ago proteins exclusively use ssRNA guides” [2]; “Eukaryotic Argonautes bind small RNAs and use them as guides to find complementary RNA targets and induce gene silencing” [1]; “several prokaryotic Agos, unlike their eukaryotic homologs, have the remarkable ability to bind single-stranded DNA guides *in vitro* and are capable of utilizing them for cleavage of RNA targets” [4]; “subsequent structural work with eukaryotic Argonautes continued to bolster the RNA guide–RNA target model” [5]; “the ability of eukaryotic Argonautes to incorporate ssRNAs as guide molecule is a universal activity that was inherited from the primordial ancestral Argonaute protein” [6].

These are not merely statements of scientific consensus regarding the nature of guides for eukaryotic Ago, but also serve rhetorical functions. For example, they heighten the contrast between mammalian and prokaryotic Argonautes, and emphasize the importance and novelty of the DNA interference phenomenon. Moreover, they also have the unintended effect of warning away readers who might naively wonder whether eukaryotic Ago proteins are also capable of interacting with DNA in any biologically relevant fashion at all. Like a policeman, these statements say: “Move along, folks, show’s over; nothing to see here. Move along quickly now!”

### Evidence for interactions of Argonaute with DNA and DNA-like nucleic acids

Over the past decade, eukaryotic Argonaute proteins have shown a steady increase in the number of its well-documented protein and RNA binding partners, its subcellular locales and its biological functions [6,7]. Not only does Ago interact with a variety of classes of short RNAs [6], it binds longer structured ncRNAs such as pre-miRs and certain tRNAs, as well as single stranded RNAs [8,9]. In fact, Ago can bind directly to mRNAs [10] and can inhibit protein translation even in the absence of RNA guide strands, e.g. when tethered to the mRNA [11] or when using Smaug as a protein-based guide [12].

It is not absurd to wonder if eukaryotic Ago might interact with DNA as well, since isolated domains of Argonaute proteins do bind DNA in the test tube. For example, the human Ago2 MID domain, which binds to the 5′ end of small RNAs, shows no strong binding preference towards the sugar conformation in the nucleic acids. RNA and DNA have comparable dissociation constants (Kd = 35 μM for DNA, 53 μM for RNA) [13]. In addition, the crystal structure of human Ago2 reveals that it does not have any direct hydrogen bonds to the 2′ hydroxyl groups of the guide strand for RNA recognition, which may explain why DNA bases and 2′ fluoro substitutions are well tolerated in the antisense strand of siRNAs [14]. *Drosophila melanogaster* Ago2 has similar binding affinity to 21nt ssDNA and ssRNA of the same sequence, and recognizes 21 bp dsDNA [15,16]. The PAZ domain of *D. melanogaster* Ago1 shows binding to 26 nt ssDNA, albeit with lower affinity than to the equivalent ssRNA sequence [17].

Argonaute proteins also bind DNA-like nucleic acids. Experiments using 21 nt siRNA with substitutions of 2′-fluorocytidine and 2′-fluorouridine in the antisense strand showed only slight decrease in RNAi in HeLa cells when compared to control siRNA, showing that the 2′-OH is not needed for RNAi [18]. Further modifications of the antisense strand with inclusion of deoxyguanine and deoxyadenine also do not affect RNAi significantly. However, replacing either the entire antisense strand or both strands with DNA abolishes RNAi completely [18]. DNA-modified-siRNA-dependent gene silencing requires TRBP2 and PACT, which bind to dsRNA, but not normally to dsDNA [19]. This might be one reason why, even though Ago2 may be able to bind ssDNA/dsDNA, DNA-guided RNA interference is not strongly induced.

### siDNAs

In contrast to the negative finding of Chiu and Rana [18], three articles report that double-stranded DNA (siDNA) can silence viral and cellular RNAs in living cells. a) Lamberton and Christian [20] compared 21 bp siRNAs vs. siDNAs and found that both caused significant silencing of its target (G6PD) in a sequence dependent manner in CHO cells, with similar kinetics, although siDNAs produced less inhibition at equal doses [20]. b) Nowak and coworkers reported that double-stranded DNA (siDNA) is able to reduce expression of a viral RNA in tobacco plants, whereas a scrambled dsDNA sequence is inactive. In human HeLa cells, siDNA reduced expression of a co-transfected reporter RNA by 50% and 78%, using 25nM and 100nM of siDNA, respectively (though they did not establish whether the effects of siDNA in HeLa cells are sequence-specific) [21]. siDNA is less potent than siRNA, which reduced expression by over 80% at only 5nM [21]. Although the role(s) of Ago in these siDNA effects have not been investigated, they do provide possible examples in which DNA might act as a guide for Ago and deserve further scrutiny. c) Finally, Moelling et al. [22] employed a partially double-stranded DNA oligonucleotide to target and cleave a particular viral RNA sequence in HEK293 cells. In this paradigm, cellular RNAse H appears to mediate the cleavage of target RNA within the DNA/RNA hybrid under normal conditions. However, when RNAse H is knocked down and Ago2 is overproduced via transfection, then Ago2 protein could also mediate cleavage of the RNA target (presumably via its PIWI domain which is RNAse H-like) [22]. This example shows that Ago might potentially participate in RNA silencing of DNA/RNA hybrids, even if it does not use the DNA as a guide for target recognition.

### Co-expression of Argonaute proteins with DNA in different subcellular locales

Probably the most important reason that eukaryotic Ago-DNA interactions have not been taken seriously is that, as stated by Olovnikov et al. [1,4]: “small DNAs have not been found in association with Argonaute proteins in eukaryotes. Natural short single-stranded DNAs, to our knowledge, have not been found in any cellular pathway, though short DNA sequences, Okazaki fragments, paired with long DNA are synthesized during replication of the lagging-DNA strand.” Although no one has identified very short DNAs (~21 nt) in eukaryotes of the type expected to be guides, no one has really looked for them either! When we carried out a search of the literature, we were able to identify a number of studies that have described DNA, relatively short DNA fragments (50-500 bp), and/or single-stranded DNAs as being naturally expressed in a number of biological locales:

- DNA in the nucleus. Ago proteins are known to enter the nucleus and perform several functions there, such as directing transcription, centromere heterochromatin formation, alternative splicing, and DNA damage repair [23]. It is thought that Ago forms a complex with miRNAs and siRNAs that interact with RNAs (e.g. promoter-associated RNAs) transcribed at these sites [7]. To our knowledge, no one has investigated whether Ago binds genomic DNA directly. However, single stranded DNA may be accessible not only during replication (as mentioned above) but also at active promoters, where portions of genomic DNA are unwound and might conceivably be targeted by Ago/RNA complexes. This might regulate the rate or progression of transcription at these sites, even if the DNA is not cleaved. It has also been proposed that Ago-miRNA complexes might bind to specific double-stranded sites on promoters to form triple-helix structures [24].
- DNA in the cytoplasm. Non-mitochondrial DNA was first observed in cytoplasm over 40 years ago. After demonstrating that cytoplasmic DNA does not simply consist of nucleosomes or other fragments associated with dead or dying cells, at least two pools of cytoplasmic DNA were characterized – one associated with microsomal fractions, and one associated with the plasma membrane. Cytoplasmic DNA levels vary in a cell line-dependent fashion [25]. Further studies showed that cytoplasmic DNA represents 1-2% of total cellular DNA in myeloma cells. Structurally, more than half of cytoplasmic DNA had a sedimentation constant of 6-8S, the remaining being more heterogeneous (12S to 40S) [26]. Cytoplasmic non-mitochondrial DNA with size in the range of 50-500 bp was observed in mouse L929 and Ehrlich ascites tumor cells, and some cytoplasmic DNA sequences, when cloned and transferred to non-tumor cells, could immortalize recipient cells [27]. Density and cloning analyses suggest that these sequences may be partially single-stranded and/or associated with RNA or metallo-proteins [27]. Challen and Adams [28] found that a portion of cytoplasmic DNA is short (~100 nt) and single-stranded [28]. Lerner et al. [29] observed plasma membrane-bound cytoplasmic DNA accounted for 0.5% of total DNA content in continuously growing human diploid lymphocytes (WIL2 cells) [29]. They found that more than 99% of the DNA molecules are linear and appear to be oriented with respect to the membrane. More recently, deep sequencing revealed that this DNA originates mostly from centromeric and pericentromeric chromosomal regions [30], with particularly strong enrichment in 171-bp α-satellite repeats (ALRs) that are transcribed by co-localized Pol II. Some studies of microsomal-associated DNA also found that it consisted predominantly of repetitive sequences [31].

Many cell types have a surveillance system that detects the presence of cytoplasmic DNA (as well as dsRNA), which can activate an innate immune signaling cascade that involves the production of type I IFN [32–34]. Certainly, cytoplasmic DNA can be induced under pathological conditions such as viral infection, inflammation and oxidative damage, but these arise from different sources than naturally occurring cytoplasmic DNA [35–37], and it is not clear whether the latter would necessarily elicit any signaling response under normal conditions. (Note that similar worries had been raised in objection to the presence of naturally occurring dsRNA in cytoplasm, yet siRNAs and longer RNA hybrids are both naturally expressed in situations that do not elicit an interferon response (reviewed in [38,39]).

Interestingly, DNA with features similar to cytoplasmic DNA was found to be extruded from living cells in association with RNA, proteins and lipids; this DNA is relatively resistant to DNAse, and could be taken up by recipient cells [40]. The extracellular DNA complexes are described as smaller and having lighter buoyant density than microvesicles and exosomes (see below), and may be more akin to lipoprotein particles and other nucleoprotein complexes shed from cells, some including Argonaute proteins, which are known to bind miRNAs and transport them to recipient cells [41]. To our knowledge, no one has immunopurified Ago proteins from either cytoplasm or conditioned cell medium to learn if Ago is associated with DNA.

c) DNA in extracellular vesicles (microvesicles (MVs) and exosomes). Argonaute proteins are components of microvesicles and exosomes, and have been reported to bind to small RNAs expressed therein [42,43]. Exosomes express a mix of miRNAs and other small RNAs, longer ncRNAs and mRNAs which appear to be transferred to recipient cells, and may be translated or regulate translation within recipient cells [44–46]. DNA is well characterized as a component of prokaryotic outer membrane vesicles (OMVs; see Endnote^a^) and a variety of reports indicate that DNA is also contained within mammalian extracellular vesicles:

Prostate cells secrete vesicles called prostasomes that contain DNA fragments from ~5 kb up to 13 kb; these are derived from the entire chromosomal genome [47]. Prostasomes are able to fuse with sperm cells, introducing exogenous DNA and RNA into sperm cells [48–50]. Human vascular smooth muscle cells also release extracellular vesicles containing dsDNA (1-20 kb) that is taken up, localized in the nucleus and translated into functional protein by other cells [51]. Vesicle populations are not homogeneous regarding size, protein composition, buoyant density and electron density, suggesting that the DNA content might also be heterogeneous [52]. In fact, DNA immune-labeling studies showed that only about 10% of the exosomes released by B16-F10 murine melanoma cells carry detectable DNA [53].

Human cancer cells release vesicles that contain genomic DNA, sometimes in much higher amounts than normal cells. Balaj et al. [54] reported that MVs from cancer cells overexpress c-Myc gene sequences and have elevated levels for both RNA and DNA (8- to 45-fold and 10- to 25-fold, respectively) when compared to normal fibroblasts [54]. Exosomal DNA content is 20-fold higher in tumor exosomes than in two normal stromal fibroblast lines and exosome-derived DNA abundance varies widely across cancer cell lines [53]. Single-stranded DNA is expressed in glioblastoma and medulloblastoma cell-derived MVs and far more abundant than in normal cells [54]. Inhibition of DNA replication with mimosine, an alkaloid that arrests the cell cycle in late G1 phase, before DNA replication [55], results in a decrease of 50% in DNA yield from MVs, raising the possibility that some of the ssDNA may be fragments generated during DNA replication and mitosis [54]. Alternatively, some of the exosomal DNA may be derived from reverse transcription of cellular RNAs, as supported by the high reverse transcriptase activity in tumor-derived microvesicles [54].

In contrast to Balaj et al [54], Thakur and coworkers showed that DNA associated with several types of tumor-derived exosomes was digested by dsDNA-specific DNase, but not by ssDNA nuclease, indicating that most of the exosomal DNA is double-stranded [53]. Sequencing of murine melanoma-derived exosomal DNA revealed that it derived from the entire genome in an unbiased manner, representing sense and antisense strands of gene-coding regions, as well as intergenic regions; no fragments were significantly enriched or depleted and no mitochondrial DNA was detected [53]. Similarly, exosomes derived from pancreatic cancer cell lines, as well as from pancreatic cancer patients’ serum, contain large dsDNA fragments of more than 10 kb that uniformly mapped back to all chromosomes [56].

Not all extracellular DNA is vesicular, and some may derive from necrotic and apoptotic cells, which may complicate analyses if not taken into account. However, all the studies reviewed here performed some sort of treatment to the extracellular vesicles prior to DNA extraction in order to diminish contamination by externally-bound DNA. The most common procedure is DNase treatment, which presumably degrades unprotected DNA [47,54,56,57]. An elegant method used by some for determining the origin of exosomal DNA is treating exosomes with two different DNA-staining dyes, one that can permeate the membrane and another that cannot (acridine orange and propidium iodide, respectively). For both prostasomes and cardiomyocyte-derived microvesicles, flow cytometry analysis showed that vesicles treated with acridine orange exhibited enhanced fluorescence, while the ones treated with propidium iodide had florescence signals compared to untreated control. This implies that the DNA is in fact inside the vesicles, not simply attached to the membrane from the outside [47,52].

It is interesting to note that both cytoplasmic DNA and extracellular vesicle DNA are expressed at greatly elevated levels in cancer cells relative to normal cells. In such a pathological scenario, if not under normal conditions, there may be appreciable binding of Ago to DNA despite its lower affinity compared to RNA. If so, DNA might displace Ago from binding to its normal partners (miRNAs, siRNAs and other short ncRNAs), disrupting tightly controlled post-transcriptional gene silencing processes that are vital to correct functioning of a normal cell. The possible contribution to cancer pathogenesis is a strong motivator for further investigation of Ago-DNA binding.

## Conclusion

The current consensus of the RNA field is that eukaryotic Argonaute proteins employ RNA guides and target other RNAs. The alternative -- that eukaryotic Ago has biologically important interactions with DNA – has not been seriously considered, in part because the only role contemplated for DNA was as a guide strand, and in part because it did not seem plausible that any natural source of suitable DNAs exists in eukaryotic cells. When we took a peek into the literature in search of potential DNA binding partners for Ago, we found an assortment of odd characters worthy of the Exploding Plastic Inevitable, whose very existence (let alone possible functions) are poorly characterized and hard to fit within existing scientific frameworks: siDNAs; single-stranded DNAs residing in extracellular vesicles; and satellite-repeat DNA fragments that are associated with the plasma membrane and transcribed by Pol II, to name three. If Ago were to be shown to bind to any of these forms of DNA in vivo, especially in states of cancer, this would go a long way towards integrating these entities into the mainstream. Therefore, we predict that the field of eukaryotic (particularly mammalian) Ago-DNA binding and functions, which currently does not exist at all, has the potential to rise and become prominent in the near future.

### Implications for the informatics of scientific discovery

A second underlying purpose in writing the present article is to evaluate the literature surrounding a specific situation in biology, with an eye towards understanding the needs and requirements for developing new informatics tools that can assist investigators in scientific discovery [58–61]. The situation of negative consensus – that is, the consensus of a field that a given phenomenon does NOT occur – would seem to be quite common in science. Yet to our knowledge, no one has studied how often it occurs, nor has it been emphasized as a pivotal “weak link” in the chain of existing knowledge. There is no search tool to help scientists identify scientific statements that reflect a negative consensus, nor to help them assess whether the negative consensus has been reached due to (vs. in the absence of) direct, intensive experimental testing. One possible way forward is to undertake text mining of BioNØT, a database of negated sentences taken from the biomedical literature [62]. Negative statements that occur repeatedly in the literature may be regarded as candidates to reflect scientific negative consensus, and can be further investigated in their original contexts. We hope that further analyses of the negative consensus can assess its value generally in identifying potential new promising lines of investigation before they begin to emerge.

The Arrowsmith two-node search tool [59,60] is designed to identify items or concepts that can bridge two fields of study in a meaningful way, especially if the two fields are apparently disparate and have little direct overlap in terms of shared articles, authors or citations. However, the present case study is not ideal for analysis by Arrowsmith, for several reasons. The two fields “eukaryotic (or mammalian) Argonaute” and “DNA” are far from disparate; furthermore, the tool is intended to be applied to topically focused literatures [59], whereas DNA is a voluminous and far-ranging literature. One of the potential bridging terms that we identified by manual searching in the present case is “cytoplasmic” – but this is rather nondescript and general, and the Arrowsmith tool assigns it an estimated probability of relevance of 0.0 that would not invite further scrutiny.

PubMed and other information retrieval tools can find articles that explicitly discuss “Argonaute AND DNA” together in the same article, and arguably, one can rely on recent review articles to summarize the predominant themes, core knowledge and obvious gaps that call for further research. However, there is no search tool to help scientists characterize the “penumbra” of any given scientific question – that is, to collect and sort through the findings that are untidy, incidental, neglected, un-replicated, uncited, and/or otherwise not fitting inside the mainstream areas of study. Refs. [21,22,25], cited above, among others e.g., [63], can be considered to fall within the penumbra of the mammalian Argonaute field. It may be an interesting question to examine whether different scientific questions vary in the relative size and nature of their penumbras, and if so, whether characterizing the penumbra gives insight into features such as the growth of the field or rate of new discoveries (e.g., number of patents awarded).

## Endnote

^a^Most Gram-negative bacteria and some Gram-positive bacteria secrete outer membrane vesicles (OMVs), which are similar to exosomes in size and which contain phospholipids, proteins, and lipopolysaccharides as well as DNA [64,65]. DNA in OMVs is generally thought to be double stranded, derived from both plasmids and chromosome, and varying from ~500 bp to more than 3 kb [66,67]. In some species OMVs can release their contents inside targeted bacteria [68,69]; reviewed in [64]. There are broad similarities between eukaryotic and prokaryotic vesicles as mediators of inter-cellular communication [70,71], and as just reviewed, both mammalian vesicles and bacterial OMVs can transfer DNA to recipient cells. There are broad similarities between eukaryotic and prokaryotic vesicles as mediators of inter-cellular communication [70,71], and as just reviewed, both mammalian vesicles and bacterial OMVs can transfer DNA to recipient cells. Yet to our knowledge, no one has investigated further parallels, such as whether OMVs express Argonaute proteins (or even if they express specific RNAs). Nor has anyone asked whether OMVs express short DNAs that might potentially serve as guides for prokaryotic DNA interference. For example, no one has examined OMVs to see if they express Argonaute proteins (or even if they express specific RNAs). Nor has anyone asked whether OMVs express short DNAs that might potentially serve as guides for prokaryotic DNA interference.

## Reviewers’ comments

### Reviewer’s report 1: Eugene Koonin, National Center for Biotechnology Information

This is an exceptionally lively article that challenges the common wisdom that eukaryotic Argonaute proteins can only use small RNA guides to cleave or regulate other RNAs. The recent demonstration that some of the prokaryotic Argonautes employ DNA guides prompts the authors to consider various scenarios under which Argonaute binding to DNA could be biologically relevant. It is particularly interesting that the amount of DNA-containing vesicles greatly increases in cancers, suggesting the possibility for important roles of Argonautes in tumorigenesis.

I agree with the authors that the apparent current consensus on the exclusive RNA utilization of eukaryotic Argonautes, despite the available biochemical data on DNA-binding, is based more on non-critically accepted dogma than on solid negative results. The possibility of DNA-related functions of eukaryotic Argonautes is important and certainly merits experimental investigation.

I should note that this article reads to me more like Hypothesis than a Comment. Furthermore, in my view, the structuring of the abstract into Background, Results and Conclusions is slightly misleading. The paper does not really report results but a hypothesis, so either an unstructured abstract or one divided into Background, Presentation of the hypothesis and Testing the hypothesis, would be more appropriate.

Authors’ response: In the revised version of this manuscript, we have created an unstructured abstract. The reasons why we prefer the Comment format rather than Hypothesis are discussed following Dr. Makarova’s review.

### Reviewer’s report 2: Kira S. Makarova, National Center for Biotechnology Information

Drs. Smalheiser and Gomes described a hypothesis suggesting that in pathological conditions specifically in cancer cells short DNAs might displace normal RNAs associated with of eukaryotic Argonauts and thus disrupt important regulatory networks. In principle this is a non-controversial and falsifiable hypothesis and in a sense an example of the thinking outside the box. This being said along with several methodological and philosophical issues of scientific research mentioned throughout the text of the paper there is yet another one - the absence of an objective way to assess the strength of a hypothesis and thus judge if the hypothesis is worth an attention of the wide scientific audience. Often an “interesting” hypothesis relies on the statements/observations the connection between which is not direct and obvious otherwise it becomes trivial. Here for example authors link the presence of elevated amounts of the DNA in cancer cells and ability of Argonauts to bind DNA. These are two indirect observations and connection between them is not apparent. Back to the hypothesis strength. In my opinion it is generally determined by two criteria: 1) how detailed and mechanistically elaborated the hypothesis is and 2) how likely are alternative hypotheses/explanations of the observations which is in turn directly related to the complexity of the phenomenon in question (if it is complex there is a large network of observations any subset of which can be logically connected and can be equally likely). It is quite clear that cancer pathogenesis is extremely complex phenomenon with numerous changes in many cellular processes and molecular interactions not mentioning that there are different types of cancers that have considerably different phenotypes. At least several different cancer agents are known and therefore we may expect that molecular processes leading to the pathology would be quite different too. Since it is complex it is quite easy to build numerous hypotheses explaining any part of the phenomenon (with and without involvement of Argonaut DNA or RNA) and even find the considerable amount of the literature that reports the observations of a choice. So with regard of this criteria the hypothesis appears to be weak.

Now the mechanistic details. Authors suggest a very simple mechanism of Argonaut involvement --DNA replaces RNA and Argonaut stops regulating gene expression in a proper way. This is an absolutely realistic mechanism and authors present quite a detailed and convincing analysis with many evidence from the literature suggesting that DNA which Argonaut can potentially bind is present in both normal and cancer cell. However it appears that DNA does not displace RNA in Argonauts (since their normal function involve RNA species) in normal cells and all the evidence which is relevant to the cancer cells mentioned in the text seems to come from the cell that are already seriously and irreversibly damaged and it does not matter in this case if Argonaut binds DNA or not too many things went wrong already. So the main question is if the DNA accumulation capable to disrupt normal Argonaut function occurs before or after irreversible damage of other cell systems caused by malfunctioning of other cell components. Is there any data on dynamics of key components (including relevant RNA) involved in the proposed mechanism along with indicators of the disruption of normal cell function? Even if a lot of DNA present in the cytoplasm are there any indications that it is accessible for Argonaut and not bound already by numerous DNA binding proteins? These are important details that if supportive could make the hypothesis stronger.

Authors’ response: We are grateful to receive the comments of Dr. Makarova (and Koonin), who are truly knowledgeable and appropriate reviewers for this article. In the revised version of this manuscript, we have expanded the discussion of more general issues related to scientific hypothesis formation and assessment which underlie and drive this article. It is important to note that we view this article not simply as presenting a specific hypothesis (Ago binds DNA in cancer cells), which would be best categorized as a Hypothesis article, but as presenting a case study of a scientific question which is in a state of “negative consensus”. We explore the reasons for this negative consensus, demonstrate the lack of actual experimental data supporting the negative consensus, and carry out a far-reaching manual search of the literature to uncover circumstantial evidence that makes the possibility of Ago binding DNA at least plausible and worth exploring, in a variety of biological contexts. All of these analyses are relevant to our article. In this regard, the revised article points out explicitly what was previously only hinted at: that the far-ranging and rather open-ended literature searches constitute raw material for studying how to create new informatics tools that can assist investigators in the future.

Dr. Makarova has raised a number of potential objections and pointed out potential weaknesses of the notion that Ago might bind DNA in cancer cells. We agree that such cells are subject to multiple (and progressive) alterations in signaling pathways and genome modifications, and it is uncertain what effects would be produced by altering Ago-RNA interactions in cells that are already abnormal. On the other hand, cancer cells are not all that seriously and irreversibly damaged -- they are still able to grow, reproduce, secrete, migrate, metastasize, and so forth despite expressing high levels of cytoplasmic and exosomal DNA. Therefore, it is certainly worth exploring whether altering Ago-RNA interactions is an important step in cancer pathogenesis. We agree that current knowledge does not allow us to say whether cytoplasmic DNA is accessible for binding to Ago, but this weakness can be “cured” by investigating further. Without formulating the hypothesis in the first place, there would be no stimulus to finding out. Going further, we agree that current knowledge does not provide the expectation that Ago binds DNA under physiological conditions in “normal” cells. However, let’s remember the precedent of growth factors, which were originally expected to be tumor-specific, and indeed growth factors and their receptors are often up-regulated in different types of cancer; yet later these were found to be ubiquitous regulators in cell biology. Should Ago-DNA interactions be shown to occur in cancer cells, it will be a natural progression of the scientific process to explore if this also occurs in normal cells.

### Reviewer’s report 3: Alexander Maxwell Burroughs, National Center for Biotechnology Information (nominated by L Aravind, National Center for Biotechnology Information)

Smalheiser and Gomes present a thoughtful and compelling counterpoint to the largely “silent” consensus that eukaryote PIWI/Ago modules are capable of binding to only RNA-RNA duplexes as opposed to RNA-DNA hybrid or DNA-DNA duplexes. They outline historical reasons for the lack of experimentation on such alternative substrates point to recent developments hinting at the possibility of eukaryotic PIWI/Ago DNA binding and describe several plausible substrates containing DNA that could be duplex targets of the PIWI/Ago module in eukaryotes. More broadly the authors generalize the issue as an example of an unfortunate kind of consensus-building in science one based more on established thinking and less on experimental findings.

I strongly agree this is an issue deserving of more experimental consideration and support publication of the manuscript. I have a few recommendations and suggestions which I submit could bolster/clarify the arguments advanced in the paper.

1. One source of widespread confusion in PIWI/Ago studies (both experimental and computational) is the lack of understanding of the relationships between different members of the family. The Argonaute proper subfamily of the canonical eukaryotic PIWI/Ago family is a more recent innovation in the general context of PIWI/Ago evolution. Within the ocean of eukaryotic diversity the PIWI proper subfamily is far more widely distributed and demonstrably closer to the DNA-binding prokaryotic PIWI (pPIWI) proteins. Immediately the relevance to this article is clear: despite representing a later innovation within the AGO/PIWI superfamily orthologs of Argonaute have been the subject of the bulk of existing research. While it is true that PIWI orthologs like Argonaute proper orthologs have demonstrated roles in RNA-RNA duplex binding (notably in ciliates and the basal diplomonad Giardia) and the claim cannot be made that RNA-RNA duplex binding is an evolutionary development concomitant with the emergence of the Argonaute proper subfamily what the manuscript states about the lack of exploration of alternative substrates in the Argonaute subfamily holds even more so for the PIWI subfamily the version most closely related to the DNA-associating pPIWI domains.

As the article discusses all eukaryotes and as many eukaryotic lineages lack genuine Argonaute orthologs it is necessary to mention PIWI alongside Argonaute. I would further recommend referring to the family containing the Argonaute proteins as PIWI/Ago.

2) The article could benefit from a more detailed historical perspective regarding the substrate-binding propensities of PIWI/Ago. The notion of a potential DNA-associated role for the PIWI/Ago superfamily long predates the 2009 article referenced in the paper: it was first postulated in 2000 after observing strong genome concordance between RNAi components and chromatin-modifying and splicing factors [72]. In 2005 two papers from the Patel group clearly demonstrate the preference of prokaryotic PIWI/Ago for binding DNA-RNA hybrid duplexes [73,74]. It was primarily these findings supplemented by (at the time) relatively thin genome associations which informed the predictions in the 2009 Makarova paper. Ultimately I think this further underscores the blind spot potential PIWI/Ago DNA-binding has sat in; even after clear experimental data was presented it took ~9 years before this line of research was advanced further!
3) I think the manuscript somewhat underplays support found in evolutionary reasoning for potential eukaryotic PIWI/Ago binding of DNA-containing substrates. This is also a poorly understood area in the literature as evidenced by the direct quotations provided by the authors in the Background section. Several recent findings have particular relevance to this subject and could be mentioned in the main text in support of the central contention of the manuscript.
  - The recent identification of the prokaryotic PIWI-RE family (distinct from the classical pPIWI family investigated by Makarova [3] and the subjects of all experimental testing to date including the recent PIWI-IP pulldown experiments). This family was the first prokaryotic PIWI/Ago family to be unequivocally linked via genome association with a DinG helicase and restriction endonuclease domain to RNA-DNA hybrid duplex binding [75]. PIWI-RE was speculated after further analysis to most closely approximate the ancestral PIWI module which emerged after the fusion of the MID domain to an RNaseH domain likely descending from the Endonuclease B/UvrC assemblage of RNaseH enzymes [76]. In addition an update to the Makarova [3] analysis found in the same Burroughs/Ando/Aravind paper clarified the genome associations of the pPIWI family: all of the so-called “class I” and several“class II” pPIWI proteins are tightly-linked in operons to DNA-processing enzymes; some of these associations were overlooked in the 2009 Makarova paper and some were added to genome databases in the interim. The sum of these observations unequivocally establishes that the ancestral PIWI/Ago bound DNA-RNA hybrid substrate duplexes; in other words the RNA and DNA identified in the Olovnikov and Swarts pPIWI pulldowns are not anomalies but rather represent the ancestral PIWI/Ago condition. While it appears clear that a shift genuinely occurred at some point from a preference for a RNA-DNA hybrid substrate to a dsRNA substrate this still suggests the ability to bind hybrid duplexes and to a lesser degree the potential ability to bind dsDNA duplexes may indeed be present in eukaryotes at least in certain conditions or contexts.
  - The discovery of the MedPIWI family a novel PIWI/Ago family found in the Med13 protein of the Mediator complex and conserved across almost all eukaryotic lineages (excepting kinetoplastids) is of particular relevance to this discussion. If any PIWI/Ago module in eukaryotes is a candidate to bind RNA/DNA duplexes it would likely be the one localizing to the transcriptional co-activating Mediator complex which is presumably in quite close contact with ssDNA formed in the wake of the opening of the transcriptional bubble [75]. While this certainly does not preclude the possibility that the classical eukaryotic PIWI or Ago subfamilies are capable of binding DNA MedPIWI nicely dovetails with the discussion in “DNA in the nucleus and mitochondrion” section and further evidences potential RNA-DNA duplex binding across eukaryotic representatives of the PIWI/Ago superfamily.

Authors’ response: Dr. Burroughs has done a great service by pointing out many aspects of PIWI/Ago that we did not consider in the original article. This is a rich addition and we are glad that his review will appear alongside the published article! We have appended the articles that he cited into our own reference list. However, the review makes us realize that our own scope, expertise and literature review was limited largely to mammalian systems. For example, we did not include Piwi proteins in the original article because, apart from the germline, they are relatively poorly characterized in differentiated mammalian tissues. There is not even a consensus regarding which Piwi proteins are expressed in brain, for example, or what their binding partners are, in contrast to a very extensive body of investigations of Ago isoforms. In the revised version, we have changed the word Eukaryotic to Mammalian in the title to better reflect the scope.

### Reviewer’s report 4: Isidore Rigoutsos, Jefferson Medical College

In this manuscript the authors discuss the possibility that Ago binds DNA and that these interactions are biologically meaningful. The likelihood that in certain settings Ago:DNA binding interactions could compete with or even displace Ago:RNA interactions is certainly very intriguing and warrants further exploration. The authors have carefully built an argument in support of this possibility through a search of the literature. It is rather unfortunate that the field on more than one occasion has ignored the available data, in this and in other contexts. The authors should consider including in their references two brief articles from the April 01 2010 issue of Nature entitled “Hypotheses first” and “Data first” [77,78].

Interestingly enough there are also several ‘mirror’ examples in the literature of DNA binding proteins interacting with RNA. For examples, RNA polymerase II (POLR2), SMAD1, and the ZPF36/ZPF36L1/ZPF36L2 members of the tristetraprolin family are known to be transcription factors and to bind to DNA. However some of these proteins can bind both DNA and RNA with examples going back more than 10 years (e.g. Gallia et al. NAR 2000, Krishna et al. NAR 2003, BabuRajendran et al. NAR 2012, etc.). More recently, DiRuscio et al Nature 2013 showed that DNMT1 can also bind numerous distinct RNAs.

I view all of these mirror examples as more arguments in support of the authors’ conjecture. There is a precedent of discoveries that can happen when researchers start thinking unconventionally and give more weight to the data in front of them. In that spirit, this thought provoking manuscript is a well-documented ‘call to arms’ that is bound to resonate with more than a few of the community’s practitioners.

Authors’ response: Dr. Rigoutsos situates our Comment very broadly in terms of hypothesis-driven vs. data-driven approaches to science. As suggested, we have appended the suggested citations to our paper [77,78]. It is worth clarifying, however, that our Comment is not intended as a critique or criticism of the mainstream approaches to investigating eukaryotic Ago and its functions. He also points out that Ago-DNA binding would represent just one example among many in which proteins traditionally thought of as RNA-binding interact with DNA, or DNA-binding proteins interact with RNAs. We agree e.g., [79].

## Abbreviations

Ago: Argonaute
siDNA: small inhibitory DNA
MV: Microvesicle
OMV: Outer membrane vesicle
